# The Impact of Physical Hazards on Workers’ Job Satisfaction in the Construction Industry: A Case Study of Korea

**DOI:** 10.3390/bs14121197

**Published:** 2024-12-13

**Authors:** Hyun Jeong Seo, Eun-jung Hyun, Young-Geun Yoon

**Affiliations:** 1 Department of Business Administration, College of Business Administration, Kookmin University, Seoul 02707, Republic of Korea; sincedeni@kookmin.ac.kr; 2 Department of Business Administration, College of Business Administration, Hongik University, Seoul 04066, Republic of Korea; ejhyun@hongik.ac.kr; 3 Department of Occupational Safety and Housing Management, Cyber Hankuk University of Foreign Studies, Seoul 02450, Republic of Korea

**Keywords:** job satisfaction, construction management, occupational safety

## Abstract

This study investigates the impact of workplace physical hazards on job satisfaction in the construction industry, focusing on the mediating role of mental threats and the moderating effects of perceived job quality and security. The study findings indicate that exposure to physical hazards significantly contributes to mental stress, leading to reduced job satisfaction. Importantly, a heightened awareness of physical risks amplifies the mental burden, further decreasing job satisfaction. Furthermore, the study highlights that perceived job quality and job security can buffer the negative effects of mental threats on job satisfaction, suggesting that enhancing these factors may alleviate some of the adverse impacts of physical hazards. This research provides important insights into the complex relationships between physical work conditions, psychological stress, and employee satisfaction. It emphasizes the need for construction companies to implement practices that not only reduce physical hazards but also improve perceived job quality and security to foster employee well-being. The study contributes to the literature on occupational health and safety, offering practical implications for managers and policymakers aiming to enhance job satisfaction and retention in physically demanding environments. Future research should explore the long-term effects of these relationships and how they may extend to other high-risk industries.

## 1. Introduction

The construction industry presents a work environment with significantly higher physical risks than other sectors, leading to lower job satisfaction and increased job-related stress [1,2]. Specifically, the elevated physical hazards in construction not only endanger workers’ physical safety but also adversely affect their mental health [2]. These challenges undermine both worker safety and job performance, ultimately reducing productivity. Despite advancements in safety management strategies and technologies, the accident rate in the construction industry remains persistently high, underscoring the need for a more comprehensive understanding of workplace risks [3,4,5].

The causes of occupational accidents at construction sites can be categorized into human errors and physical risks [6]. Human errors include mistakes or lapses in judgment, such as communication failures, inadequate training, and psychological stress. In contrast, physical risks refer to environmental factors, such as falls from heights, exposure to hazardous substances, and accidents involving heavy machinery. Research shows that human errors account for over 80% of occupational accidents [6,7]. Key contributors to these errors include a lack of safety knowledge, insufficient training, unsafe psychological states, and unsafe behaviors. Companies have responded by investing in safety education and health systems to address these challenges, while researchers continue to explore strategies to encourage safe worker behaviors [8,9]. Nevertheless, accidents related to human error persist, indicating the need for innovative approaches.

In addition to human factors, physical hazards such as noise, vibration, and exposure to hazardous substances also contribute to job stress and unsafe behaviors among construction workers [6]. While these physical risks are well documented, their direct relationship to job stress and satisfaction remains underexplored. Most existing studies focus predominantly on human errors, leaving a gap in understanding how environmental factors impact workers’ mental health and safety behaviors. This lack of evidence may overlook critical aspects of workplace risk management.

Recently, there has been increased emphasis on managing job stress and promoting safety behaviors to reduce occupational accidents in industrial organizations [3,10]. Construction workers, frequently exposed to physical hazards, are particularly vulnerable to job stress. Risk factors such as noise, vibration, hazardous substances, and high job demands act as stressors that hinder safety behaviors and reduce job satisfaction. Lee and Lee [3] identified these stressors as primary contributors to workplace accidents. Understanding how physical hazards influence job stress and satisfaction is crucial for developing effective prevention strategies.

This study aims to address this research gap by examining the effects of physical risks on job stress and satisfaction among Korean construction workers. Specifically, it investigates how physical risk factors contribute to job-related stress and how perceptions of job quality and security moderate this relationship. By identifying strategies to manage job stress and enhance job satisfaction, this study seeks to improve safety performance and worker well-being in the construction industry.

## 2. Literature Review and Research Hypothesis

### 2.1. Physical Hazards and Mental Health Impacts in the Construction Industry

The construction industry is widely recognized for its hazardous working conditions, which stem from intense work demands, the use of heavy equipment, and challenging outdoor environments. These physical hazards include risks such as falls from heights, exposure to hazardous substances, and machinery-related accidents, all of which pose significant threats to workers’ safety and well-being [11,12]. Continuous exposure to such risks can result in cumulative mental and physical strain, increasing job-related stress, and decreased job satisfaction [13]. Compared to other industries, physical hazards are more prevalent in construction, amplifying their impact on workers’ health and job performance outcomes [14].

Although safety management systems and training programs have been implemented to mitigate these risks, the persistently high accident rates in the construction sector indicate that existing measures may be inadequate. Previous research underscores that physical hazards are a significant contributor to adverse mental health outcomes, such as anxiety and stress, which compromise job performance and elevate the likelihood of unsafe behaviors [6,11]. Despite these findings, further investigation is required to fully understand the direct and indirect impacts of physical hazards on workers’ mental well-being and job satisfaction.

The construction industry’s inherently high-risk environment exposes workers to various dangers associated with demanding tasks, extensive equipment usage, and external conditions. These factors significantly contribute to elevated job stress, which can, in turn, threaten workers’ mental health [12,13]. Studies have demonstrated that continuous exposure to physical hazards leads to anxiety and stress concerning safety, ultimately manifesting as mental threats that negatively affect work performance [14,15]. Based on these observations, we propose the following hypothesis:

**Hypothesis** **1.** *Higher levels of workplace physical hazards are positively associated with increased mental threats from job* *-related stress among construction workers.*

### 2.2. Physical Hazards and Job Satisfaction of Workers in the Workplace

Mental threats, defined as the anxiety and stress arising from job-related risks, play a pivotal role in shaping workers’ perceptions of their jobs. In high-risk industries such as construction, prolonged exposure to physical hazards can severely compromise workers’ mental health, leading to heightened levels of stress and anxiety [14,16]. This mental distress not only undermines emotional stability but also diminishes job performance, ultimately resulting in reduced job satisfaction [15].

The negative relationship between mental threats and job satisfaction is well documented in the occupational health literature. Workers who frequently face safety-related concerns often develop negative perceptions of their work environment, which directly diminishes their overall job satisfaction [16]. Furthermore, the psychological burden associated with persistent safety-related stressors exacerbates feelings of insecurity and dissatisfaction, emphasizing the critical need to address mental threats as a core component of workplace risk management.

Physical hazards—such as exposure to noise, vibration, or hazardous substances—significantly contribute to mental threats and reduced job satisfaction. Repeated exposure to these risks intensifies anxiety and stress during job performance, fostering negative perceptions of the job due to ongoing safety concerns. These hazards degrade the quality of the work environment, which directly affects workers’ job satisfaction [15]. In industries like construction, where exposure to physical hazards is constant, prolonged physical and mental fatigue further amplify dissatisfaction.

Numerous studies have consistently highlighted the close relationship between physical hazards and job satisfaction. Higher levels of workplace risk are strongly associated with increased negative attitudes toward one’s job [4,13]. Building on this foundation, we propose the following hypothesis:

**Hypothesis** **2.** *Workplace physical hazards have a negative direct effect on job satisfaction.*

### 2.3. The Mediating Role of Mental Threats in the Relationship Between Physical Risks and Job Satisfaction

The interplay between physical hazards, mental threats, and job satisfaction has been a focal point in occupational health research. Physical hazards—such as exposure to hazardous substances, excessive noise, vibration, or the operation of heavy machinery—pose direct risks to worker safety and well-being. In high-risk industries like construction, these hazards not only are a source of physical danger but also act as significant stressors that contribute to workers’ mental strain. This dual impact makes it essential to explore how physical hazards indirectly influence job satisfaction through their psychological effects.

Mental threats, conceptualized as the stress and anxiety arising from job-related risks, play a mediating role in the relationship between physical hazards and job satisfaction. Prolonged exposure to physical hazards increases workers’ awareness of potential safety risks, fostering an ongoing state of stress and anxiety. This heightened mental strain can disrupt emotional stability, hinder cognitive focus, and diminish the capacity to manage workplace challenges effectively. Consequently, workers experiencing persistent mental threats are more likely to develop negative perceptions of their jobs and exhibit lower levels of job satisfaction [12,16].

Empirical studies underscore the importance of addressing mental threats to mitigate the adverse effects of physical hazards on job satisfaction. Research findings indicate that workers exposed to high levels of physical risk report increased mental distress, which correlates with a decline in overall job satisfaction [16]. This evidence highlights the necessity of integrating mental health considerations into workplace safety frameworks, particularly in industries where physical hazards are unavoidable.

Moreover, the indirect relationship between physical hazards and job satisfaction, mediated via mental threats, holds significant implications for workplace interventions. Effective risk management strategies should focus not only on mitigating physical hazards but also on addressing their psychological impact. Initiatives such as stress management programs, mental health support services, and clear communication of safety protocols can help reduce mental threats and enhance job satisfaction.

Based on these considerations, the mediating role of mental threats is posited as follows:

**Hypothesis** **3.** *Mental threat from one’s job mediates the relationship between workplace physical hazards and job satisfaction.*

### 2.4. Moderating Role of Perceived Job Quality and Security

Perceived job quality and job security are critical determinants of job satisfaction, particularly in high-risk industries such as construction. Job quality encompasses workers’ perceptions of the value and rewards associated with their jobs, including opportunities for personal and professional growth, recognition, and skill development [17]. High job quality fosters a sense of fulfillment and stability, which can mitigate the psychological effects of job-related stress. It equips employees with the resilience needed to manage work challenges effectively, thereby enhancing job satisfaction. Luthans and Youssef (2007) demonstrated that workers with higher job quality are more resilient to stressors, experiencing fewer adverse effects on job satisfaction compared to those with lower job quality [18].

Job security, defined as the perceived stability of employment and the continuity of economic compensation, also plays a pivotal role in job satisfaction. Workers who perceive their employment to be insecure are more likely to experience heightened work-related stress, leading to reduced job satisfaction. In contrast, a sense of job security offers psychological stability, alleviating stress and enhancing job satisfaction. Research by Greenhalgh and Rosenblatt (2010) underscores that employees with high job security are less affected by the negative consequences of job stress, highlighting its positive impact on overall satisfaction [19].

In the context of occupational hazards, perceived job quality and security extend beyond their direct influence on job satisfaction by moderating the relationship between mental threats and satisfaction. Workers exposed to physical hazards often experience mental strain, such as anxiety and stress, which undermines their job satisfaction. However, high perceptions of job quality and security can attenuate these negative effects. Enhanced job quality provides workers with resources and a sense of purpose, while job security ensures stability. Together, these factors buffer the psychological strain caused by workplace risks.

The indirect effect of physical hazards on job satisfaction via mental threats may also be moderated via perceived job quality and security. Workers with high levels of perceived job quality and security are likely to experience reduced negative impacts of mental threats, as their evaluations of job quality and security offset the strain caused by hazardous working conditions. This highlights the need to consider how job-related evaluations interact with psychological and physical stressors in shaping job satisfaction outcomes.

Building on this, it is anticipated that perceived job quality and security will moderate both the direct and indirect relationships between workplace hazards and job satisfaction. Specifically, higher levels of perceived job quality and security are expected to diminish the negative effects of mental threats on job satisfaction and reduce the mediated impact of physical hazards on satisfaction through mental strain.

The preceding discussion provides the foundation for the following hypotheses:

**Hypothesis** **4.** *Perceived job quality and security moderate the relationship between mental threats and job satisfaction, such that higher levels of perceived job quality and security attenuate the negative effects of mental threats on job satisfaction.*

**Hypothesis** **5.** *The indirect effect of workplace physical hazards on job satisfaction, mediated via mental threats, is moderated by perceived job quality and security. Specifically, higher levels of perceived job quality and security weaken the negative indirect effect of physical hazards on job satisfaction.*

These hypotheses reflect the intricate interplay between workplace hazards, psychological factors, and workers’ perceptions of their jobs. They underscore the importance of a holistic approach to workplace risk management that includes strategies to enhance perceived job quality and security alongside efforts to mitigate physical hazards and mental threats. By adopting such comprehensive measures, organizations can foster safer, more supportive work environments that promote higher job satisfaction, even in high-risk industries.

## 3. Methodology

### 3.1. Data and Sample

This study analyzed data from the 6th Korean Working Conditions Survey (KWCS) conducted between 2020 and 2021. The KWCS, a nationally authorized survey (Authorization No. 380002), follows the methodology of the European Working Conditions Survey (EWCS). The survey targets individuals who worked at least one hour during the survey period, including standard employees, unpaid family workers, workers on temporary leave, and those in special employment arrangements.

To reflect the construction industry’s unique employment structure, this study included permanent, temporary, and daily workers. This approach ensures a more representative analysis of the sector, where temporary and daily work arrangements are common. The final sample comprised 2202 construction workers, including 1373 permanent workers (62.4%), 196 temporary workers (8.9%), and 633 daily workers (28.7%).

Given the recognition that job stress and job satisfaction vary by employment type, the inclusion of both temporary and daily workers, alongside permanent workers, ensured a more representative analysis of the construction workforce. This approach allows for a comprehensive examination of the causes of mental stress among construction workers and provides a more precise evaluation of job satisfaction differences based on the characteristics of each employment group. For instance, an initial analysis of standardized mental stress scores (Z-scores: mean = 0, SD = 1) showed notable variations: permanent workers (M = −0.034, SD = 0.948), temporary workers (M = −0.021, SD = 1.086), and daily workers (M = −0.086, SD = 1.025). These differences highlight the importance of including diverse employment types in the analysis.

To control for potential confounding effects, the study incorporated several individual-level characteristics as control variables. These variables encompassed worker demographics (e.g., age, experience, and health status), training status within the past 12 months, skill matching with job requirements, task complexity, work autonomy, and supervisor support.

Cases with missing or invalid responses (responses of “Don’t know” or “Refused”) concerning key variables were excluded from the analysis. The final analytical sample was reduced to 2078 workers, representing a 94.4% retention rate from the original dataset. To further ensure data quality, additional screening criteria were applied: (1) Cases with extreme or unreliable responses concerning physical hazard measures (values ≥8 for any of the nine physical hazard items) were excluded to mitigate potential reporting bias. (2) Cases where respondents selected “Not applicable” (value = 7) for critical workplace social support and stress measures were removed to focus on relevant and interpretable responses.

These data cleaning procedures ensured that the final analytical sample reflected valid, reliable, and interpretable response patterns. The resulting sample size of 2078 workers, with which we examined the relationships between workplace physical hazards, mental threats, and job satisfaction in the construction industry.

### 3.2. Measures

#### 3.2.1. Dependent Variable: Job Satisfaction

We measured job satisfaction using a single-item question: “On the whole, are you very satisfied, satisfied, not very satisfied or not at all satisfied with working conditions in your job?” Responses were recorded on a 4-point Likert scale. To facilitate interpretation, we reverse-coded the variable so that higher scores indicate greater satisfaction (1 = not at all satisfied; 4 = very satisfied). While single-item measures involve limitations, they have been found to be valid in measuring overall job satisfaction.

While single-item measures face limitations, they have been widely used and validated in major national workforce surveys, including the EWCS, from which the KWCS was adapted. This measure captures overall job satisfaction, rather than satisfaction with specific job facets, aligning with our study’s focus on general workplace conditions and their broad impacts on worker well-being.

#### 3.2.2. Independent Variable: Workplace Physical Hazards

We created this measure as an average value from nine items assessing exposure to various physical hazards in the workplace. The items included the following:

A. Vibrations from hand tools, machinery, etc.
B. Noise so loud that you would have to raise your voice to talk to people.
C. High temperatures that make you perspire even when you are not working.
D. Low temperatures, whether indoors or outdoors.
E. Breathing in smoke, fumes, powder, or dust.
F. Breathing in vapors, such as those from solvents and thinners.
G. Handling or being in skin contact with chemical products or substances.
H. Tobacco smoke from other people.
I. Handling or being in direct contact with materials that can be infectious.

For each item, responses were recorded on a 7-point scale (1 = “All of the time”, 2 = “Almost all of the time”, 3 = “Around 3/4 of the time”, 4 = “Around half of the time”, 5 = “Around 1/4 of the time”, 6 = “Almost never”, and 7 = “Never”). Items were reverse-coded so that higher scores indicate greater exposure to physical hazards, and then they were averaged to create a composite measure. Cases with responses of 8 (“Don’t know/no opinion”) or 9 (“Refused”) were excluded from the analysis.

Responses were measured on a 7-point Likert scale, where 1 indicated exposure “All of the time”, and 7 indicated “Never.” To facilitate interpretation, we reverse-coded these items so that higher scores indicate greater exposure to physical hazards.

The Cronbach’s alpha for this scale was 0.89, indicating high internal consistency reliability.

#### 3.2.3. Mediator: Mental Threat from Work

We derived this measure from a principal component analysis (PCA) of five items related to work–life balance and job-related stress. The items, translated from Korean, were as follows:

A. I worry about work continuously, even when I am not at work.
B. I am too tired after work to do some of the necessary household jobs.
C. My job prevents me from giving the time I want to my family.
D. I find it difficult to concentrate on my job because of my family responsibilities.
E. I have found it difficult to fulfill my family responsibilities because of the amount of time I spend on my job.

Responses were recorded on a 5-point Likert scale (1 = “Always”, 2 = “Most of the time”, 3 = “Sometimes”, 4 = “Rarely”, and 5 = “Never”). Cases with responses of 7 (“Not applicable”), 8 (“Don’t know”), or 9 (“Refused”) were excluded from the analysis. Items were reverse-coded so that higher scores indicate a greater mental threat.

The principal components analysis using varimax rotation yielded a single dominant factor explaining 63.45% of the total variance (eigenvalue = 3.17250). The factor loadings showed strong construct validity across all items. The scale showed high internal consistency (Cronbach’s α = 0.85).

#### 3.2.4. Moderator: Perceived Job Quality and Security

We derived this measure from a PCA of seven items related to work practices and organizational support. The items assessed the following:

A. Considering all my efforts and achievements, I feel I am compensated fairly for my work.
B. My job offers good prospects for career advancement.
C. I receive appropriate recognition for my work contributions.
D. I experience strong competition with others in my workplace and for work opportunities.
E. The organization I work for motivates me to perform at my best.
F. I am concerned about potentially losing my job within the next 6 months.
G. If I were to lose or leave my current job, I am confident I could easily find a position with a comparable salary.

Responses were recorded on a 5-point Likert scale. The PCA resulted in two factors explaining 63.25% of the variance. We used the first factor, which explained 41.22% of the variance, as our perceived job quality and security measure. Factor loadings for this first factor ranged from 0.46 to 0.86, with Items 1–3 loading particularly strongly (0.84, 0.82, and 0.86, respectively). The Cronbach’s alpha for the items loading on this factor was 0.82, indicating good reliability.

#### 3.2.5. Control Variables

We measured work practices and organizational support by including seven items related to job performance and organizational support. These items were derived through principal component analysis (PCA).

In this study, several control variables were included to account for potential confounding effects. Control variables play a crucial role in mitigating the influence of external factors that could affect the research results. The specific control variables included were as follows:

A. Training: a binary variable indicating training receipt in the past 12 months (1 = yes; 0 = no).
B. Task complexity: measured through the KWCS item “Does your job involve complex tasks?” (1 = yes; 0 = no).
C. Skill match: from the KWCS item assessing skills–duties correspondence (3-point scale: 1 = need more training, 2 = skills match well, and 3 = can handle more demanding duties).
D. Autonomy: based on the KWCS item regarding the ability to decide by oneself (1 = yes; 0 = no).
E. Support from boss: from the KWCS item measuring the frequency of supervisor help/support (5-point scale from “Always” to “Never”).
F. Demographics: gender (1 = male; 2 = female) and age (continuous in years).
G. Cases with “Don’t know” (8) or “Refused” (9) responses were excluded from the analysis.

These control variables helped account for various external factors that could influence the outcomes of our research, enabling us to better understand the relationships between the key variables we studied.

### 3.3. Analytical Approach

We employed structural equation modeling (SEM) with maximum likelihood estimation and bootstrapping (200 replications) to test our hypotheses. This approach allowed us to simultaneously estimate the direct and indirect effects in our moderated mediation model while accounting for measurement error. We used standardized coefficients to facilitate the comparison of effect sizes across variables measured on different scales. The bootstrapping procedure provides more robust standard errors and confidence intervals, particularly for indirect effects in mediation analyses.

## 4. Results

### 4.1. Preliminary Analyses

Table 1 and Table 2 present descriptive statistics and correlations for study variables. Mean job satisfaction was 2.76 (SD = 0.54) on a four-point scale. Physical hazards averaged 2.16 (SD = 0.99) on a seven-point scale. Mental threat varied by employment type: permanent workers (M = −0.034, SD = 0.948), temporary workers (M = −0.021, SD = 1.086), and daily workers (M = −0.086, SD = 1.025).

Job satisfaction correlated negatively with physical hazards (*r* = −0.27, *p* < 0.01) and mental threat (*r* = −0.13, *p* < 0.01). Physical hazards and mental threat showed a positive correlation (*r* = 0.14, *p* < 0.01). Perceived job quality and security correlated positively with satisfaction (*r* = 0.44, *p* < 0.01).

### 4.2. Direct Effects on Mental Threat from Work

Table 2 presents the correlation matrix for the variables used in this study. Each cell represents the correlation coefficient between the respective pair of variables.

Our findings demonstrate that workplace physical hazards significantly increase the perceived mental threat from work (*β* = 0.135, *p* < 0.001), supporting Hypothesis 1. This suggests that construction workers exposed to higher levels of physical hazards in their work environment are more likely to experience a heightened sense of mental threat. The results highlight the psychological impact of physical risks, showing that such hazards not only pose a direct physical safety threat but also contribute to increased psychological stress and anxiety among workers. Therefore, we should view the presence of physical hazards as both a potential cause of physical injury and a source of psychological strain, emphasizing the need to address both aspects of well-being in high-risk work environments.

Task complexity shows a significant negative relationship with perceived mental threat (*β* = −0.049, *p* < 0.05). As task complexity increases, the perception of mental threat decreases. This suggests that more challenging or stimulating tasks may help alleviate stress by engaging workers cognitively. Our findings imply that tasks that provide intellectual stimulation or a sense of accomplishment can serve as coping mechanisms, reducing the psychological burden associated with one’s job. Such tasks may offer workers a sense of focus or mastery, potentially mitigating stress levels, especially in high-demand environments.

Similarly, support from supervisors is significantly negatively associated with mental threat (*β* = −0.063, *p* < 0.01). Higher levels of supervisor support correlate with lower levels of perceived mental threat. This indicates that effective leadership and supportive management practices play a crucial role in reducing psychological stress among construction workers. When workers perceive that they have adequate support, guidance, and resources from their supervisors, their psychological well-being improves, helping to buffer the negative effects of job-related stress. This finding underscores the importance of managerial practices in creating a work environment that fosters both physical and mental health.

### 4.3. Direct Effects on Job Satisfaction

Our analysis reveals that workplace physical hazards have a significant negative direct effect on job satisfaction (*β* = −0.229, *p* < 0.001), thereby supporting Hypothesis 2. This result indicates that higher exposure to physical hazards in the workplace directly correlates with lower levels of job satisfaction among construction workers. The negative impact of physical hazards on job satisfaction likely arises from the discomfort, safety concerns, and stress that workers experience due to these hazards. The presence of physical risks in the work environment fosters a sense of insecurity and unease, which diminishes workers’ overall perception of their job quality. In high-risk environments like construction sites, the constant awareness of potential physical dangers may overshadow the positive aspects of one’s job, leading to a decline in workers’ satisfaction.

Additionally, we found that mental threat from work has a significant negative effect on job satisfaction (*β* = −0.084, *p* < 0.001), which provides empirical support for Hypothesis 3. This suggests that, as workers perceive greater levels of mental threat, their job satisfaction decreases. Our findings emphasize the crucial role psychological stress plays in shaping workers’ overall attitudes toward their jobs. Mental threats, arising from job demands, work pressure, or interpersonal conflicts, can significantly diminish workers’ emotional attachment to their roles. When workers feel mentally threatened or overwhelmed, this feeling erodes their sense of fulfillment and well-being, further reducing job satisfaction. These findings highlight the need to address both the physical and psychological aspects of the work environment in order to take a holistic approach to worker satisfaction.

When examining the control variables, we found that support from supervisors is positively and significantly associated with job satisfaction (*β* = 0.178, *p* < 0.001). This indicates that greater support from management and supervisors enhances workers’ satisfaction with their roles. Supervisors who offer guidance, resources, and emotional support help create a positive work environment that boosts employee morale and satisfaction. This finding underscores the importance of effective leadership and management practices in improving job satisfaction, particularly in high-pressure and potentially hazardous work settings.

In contrast, we observed a negative relationship between age and job satisfaction (*β* = −0.064, *p* < 0.01). Specifically, older workers tend to report lower levels of job satisfaction compared to their younger counterparts. This may be due to various factors, including the increasing physical and mental demands of the job as workers age, or older workers’ potentially differing expectations and career priorities. The finding suggests that age can influence how workers perceive their job experience, with older employees possibly feeling more fatigued or less satisfied as a result of long-term exposure to work-related stressors.

Furthermore, gender showed a small but statistically significant effect on job satisfaction (*β* = 0.043, *p* < 0.05). Specifically, female workers reported slightly higher levels of job satisfaction compared to male workers. This finding may reflect gender differences in job expectations, coping mechanisms, or workplace dynamics. While the effect size is small, female workers may have a more positive perception of their jobs, potentially influenced by factors such as workplace culture, interpersonal relationships, or access to support resources.

### 4.4. Mediation Analysis

Table 3 presents the results of the moderated mediation model used in this study, showing the effects of various predictors on “Mental Threat” (the mediator) and “Job Satisfaction” (the outcome variable). The table displays the standardized regression coefficients (*β*) along with their standard errors (SE) for each relationship.

In order to examine our mediation hypothesis (H3), we conducted a mediation analysis to explore the indirect effect of workplace physical hazards on job satisfaction, mediated via the mental threat experienced by workers. Our analysis revealed several key findings that illuminate the complex relationship between physical hazards, mental threats, and job satisfaction.

First, we found that the pathway from workplace physical hazards to mental threats was statistically significant (*β* = 0.135, *p* < 0.001). This indicates that, as workers’ exposure to physical hazards increases, they are more likely to experience heightened levels of mental threat. The presence of physical hazards creates an environment where workers are not only concerned about their physical safety but also mentally preoccupied with potential risks, leading to increased psychological stress.

Next, the analysis revealed a significant negative relationship between mental threat and job satisfaction (*β* = −0.084, *p* < 0.001). This finding suggests that, as workers perceive greater levels of mental threat, their job satisfaction decreases. The mental threat, which can arise from various factors such as job demands, safety concerns, or interpersonal tensions, undermines workers’ emotional well-being and reduces their overall job satisfaction. These results emphasize the importance of managing psychological stress in the workplace, as mental threat plays a crucial role in shaping workers’ attitudes toward their jobs.

In addition to these indirect effects, we observed that the direct effect of workplace physical hazards on job satisfaction remained significant, though reduced (*β* = −0.229, *p* < 0.001). This indicates that, while physical hazards directly impact job satisfaction negatively, part of this effect is mediated via the increased perception of mental threat. Specifically, physical hazards not only reduce job satisfaction by creating a dangerous or uncomfortable work environment but also indirectly diminish job satisfaction via heightening psychological stress, which, in turn, lowers overall satisfaction.

Taken together, these findings provide evidence for partial mediation, supporting Hypothesis 3. In other words, mental threat partially mediates the relationship between workplace physical hazards and job satisfaction. The direct effect of physical hazards on job satisfaction remains significant, but part of their negative impact is explained by the psychological stress workers experience from these hazards. This suggests that physical hazards not only influence workers’ perceptions of safety but also contribute to psychological strain, amplifying the negative impact on job satisfaction.

The mediation analysis underscores the importance of addressing both physical and psychological aspects of the work environment to improve job satisfaction among construction workers. While mitigating physical hazards is crucial for directly enhancing job satisfaction, addressing the mental threat workers experience is equally important. Interventions aimed at reducing psychological stress—such as improving safety protocols, offering mental health support, and implementing stress management programs—can help buffer the negative effects of physical hazards on job satisfaction.

These findings suggest that organizations should focus on enhancing physical safety measures but also create an environment where psychological well-being is prioritized. By addressing both the physical and mental dimensions of the workplace, employers can more effectively enhance workers’ job satisfaction and overall well-being.

### 4.5. Moderation Analysis

Our structural equation model demonstrated an acceptable fit to the data, as indicated by the log-likelihood value of −26,526.836. The model explained 2.65% of the variance in mental threat from work (1–0.9734977) and 13.10% of the variance in job satisfaction (1–0.8690449). In our moderated mediation analysis, we examined how perceived job quality and security moderate the relationship between mental threat and job satisfaction, as outlined in Hypothesis 4, and how they influence the indirect effect of workplace physical hazards on job satisfaction through mental threat, as stated in Hypothesis 5.

Our analysis revealed that the interaction effect between mental threat and perceived job quality and security on job satisfaction was significant (*β* = 0.100, *p* < 0.001), supporting both Hypothesis 4 and Hypothesis 5. This significant interaction indicates that perceptions of job quality and security play a crucial moderating role in the relationship between mental threat and job satisfaction. Specifically, when workers perceive higher levels of job quality and security, the negative impact of mental threats on job satisfaction is diminished. In other words, workers who feel secure in their jobs and believe their work environment is of high quality are better able to mitigate the harmful effects of mental threats on their job satisfaction. These findings highlight the buffering role of job quality and security, suggesting that positive perceptions of job stability and work environment can protect workers’ job satisfaction, even in the presence of mental threats arising from workplace physical hazards.

Additionally, our results strongly support Hypothesis 5, which proposes that the indirect effect of workplace physical hazards on job satisfaction, mediated via mental threat, is moderated via perceived job quality and security. The analysis showed that higher levels of perceived job quality and security weaken the negative indirect effect of physical hazards on job satisfaction. Employees who perceive their jobs as secure and their work environments as high-quality are better equipped to counteract the adverse effects of mental threats on their job satisfaction.

The structural equation model demonstrated an acceptable fit to the data, with a log-likelihood value of −26,526.836. It explained 2.65% of the variance in mental threat and 13.10% of the variance in job satisfaction, indicating that the model effectively captures the psychological processes linking workplace physical hazards, mental threat, and job satisfaction.

## 5. Discussion

Our findings strongly support a moderated mediation model that links workplace physical hazards to job satisfaction through the mediating mechanism of mental threats from work, with this relationship further moderated via perceived job quality and security. These results provide valuable insights into the effects of physical work environments on employee well-being, particularly in the construction industry, and emphasize the critical role of organizational practices in mitigating workplace stressors.

First, the significant positive relationship between workplace physical hazards and mental threats (H1) underscores the psychological impact of physical working conditions. This finding corroborates and extends prior research on occupational stress, highlighting that the presence of physical hazards can induce chronic mental stress, even in the absence of actual accidents. Such persistent stress may have long-term consequences, including impaired mental health, increased burnout, and decreased productivity, consistent with the findings of Banerjee-Batist et al. (2022) [20] and Zohar (2010) [21]. In industries where physical hazards are particularly prevalent, addressing this psychological dimension is essential for safeguarding worker well-being.

Second, the negative direct effect of workplace physical hazards on job satisfaction (H2) highlights the dual impact of these hazards. Beyond immediate safety risks, physical hazards can erode job satisfaction over time. This decline may lead to broader organizational challenges, such as reduced employee retention, lower productivity, and diminished adherence to safety protocols, which aligns with the observations of Bazzoli and Probst (2022) [22]. These findings underscore the importance of implementing comprehensive workplace interventions that prioritize both physical safety and the broader psychosocial implications of hazardous work environments, as discussed by Griffin et al. (2016) [23].

The partial mediation of the relationship between workplace physical hazards and job satisfaction through mental threats (H3) offers a deeper understanding of the psychological pathways that connect working conditions to employee attitudes. This result suggests that alleviating the mental stress associated with physical hazards can enhance job satisfaction, even in settings where completely eliminating hazards is not feasible. Interventions such as counseling services, stress management training, and mental health support initiatives could be particularly effective in addressing these indirect effects. This is consistent with research by He et al. (2021) [24] and Alayyannur and Arini (2017) [25], which emphasize the importance of health as a mitigating factor in reducing the impact of physical hazards.

Most significantly, the results demonstrate the moderating role of perceived job quality and security in the relationship between mental threats and job satisfaction (H4). The buffering effect of job quality and security indicates that employees in high-quality, secure jobs are better equipped to manage the negative impact of workplace stressors. This finding aligns with the Job Demands-Resources (JD-R) model, illustrating how resources such as perceived job quality and stability can mitigate the demands posed by physical hazards and mental stress. These resources enhance employees’ sense of control and provide access to coping mechanisms, such as organizational support, training opportunities, and career development, fostering resilience and sustaining job satisfaction. Similar findings have been reported by Hofmann et al. (2003) [26] and Kim et al. (2022) [27].

## 6. Conclusions

This study makes several significant contributions to the literature on occupational health, job satisfaction, and human resource management. We provide empirical evidence on the intricate relationships between physical hazards, psychological stress, and job satisfaction in the construction industry. By integrating both physical and psychological dimensions, we emphasize the necessity of addressing these aspects simultaneously to enhance employee well-being in physically demanding jobs. Furthermore, we extend the Job Demands-Resources (JD-R) model by demonstrating that perceived job quality and security act as critical moderating resources.

From a practical standpoint, we offer actionable insights for managers and policymakers within the construction sector. While minimizing physical hazards remains a central priority, we highlight the importance of implementing strategies that enhance employees’ perceptions of job quality and security. Our findings suggest that human resource practices such as equitable compensation, career development opportunities, and transparent communication regarding safety measures can help alleviate the psychological stress associated with physical hazards. We also recommend that training programs address not only physical safety protocols but also psychological resilience, equipping workers with effective tools to manage workplace stress.

We also emphasize the need for targeted organizational interventions. For instance, by fostering a supportive work culture in which employees feel valued and secure, organizations can significantly improve job satisfaction, even in contexts where physical risks cannot be entirely eliminated. Additionally, we show that clear communication about an organization’s commitment to safety and security can reduce workers’ perceptions of risk and psychological threats.

Building on our findings, we suggest that future research identify the specific components of perceived job quality and security that are most effective in mitigating psychological threats. Longitudinal studies are needed to clarify causal relationships and explore the long-term effects of these dynamics on organizational outcomes, such as employee retention, productivity, and safety incident rates. Furthermore, we propose examining whether our findings can be generalized to other high-risk industries, such as manufacturing or emergency services, to broaden the scope of this research. Exploring individual differences, such as resilience and risk tolerance, could also provide a more nuanced understanding of how employees respond to physical and psychological stressors.

In conclusion, we shed light on the complex interactions between physical hazards, psychological threats, and job satisfaction in the construction industry. By addressing both physical and psychological aspects of workplace safety, we demonstrate how organizations can create environments that not only safeguard employees’ physical health but also promote job satisfaction and overall quality of work life. We are confident that these two approaches hold significant potential to enhance both individual well-being and organizational effectiveness over the long term.

## Figures and Tables

**Table 1:** Descriptive Statistics.

	Variables	Mean	SD
1	Job satisfaction	2.76	0.54
2	Mental threat from job	0.06	0.88
3	Workplace physical hazards	2.16	0.99
4	Perceived job quality and security	0.11	0.83
5	Training	0.17	0.38
6	Task complexity	1.54	0.56
7	Skill match	2.14	0.82
8	Autonomy	0.17	0.38
9	Support from boss	3.56	0.81
10	Gender (male = 1, female = 2)	1.11	0.32
11	Age	48.28	11.86

**Table 2:** Correlation Coefficients.

Variables	1	2	3	4	5	6	7	8	9	10	11
1	1										
2	−0.13	1									
3	−0.27	0.14	1								
4	0.44	−0.14	−0.12	1							
5	0.06	−0.03	0.03	0.08	1						
6	−0.01	−0.04	0.01	−0.12	−0.1	1					
7	−0.01	−0.02	0.04	0.02	−0.02	0.04	1				
8	0.02	0.03	0.13	0.12	0.09	−0.07	0	1			
9	0.2	−0.06	−0.06	0.37	0.11	−0.16	0.01	0.15	1		
10	0.08	0.01	−0.14	−0.02	0.01	0.02	−0.01	−0.02	−0.01	1	
11	−0.14	0.04	0.25	−0.12	−0.03	0.09	0.05	−0.01	−0.06	−0.11	1

Coefficients with an absolute value greater than 0.1 are statistically significant at the *p* < 0.01 level.

**Table 3:** Moderated Mediation Model Results.

Variables	Mental Threat (Mediator)	Job Satisfaction (Outcome)
	β (SE)	β (SE)
Workplace physical hazards	0.135 *** (0.028)	−0.229 *** (0.024)
Mental threat	-	−0.084 *** (0.020)
Perceived job quality and security	-	0.100 *** (0.026)
Training	−0.031 (0.022)	0.040 (0.022)
Task complexity	−0.049 * (0.020)	0.036 (0.022)
Skill match	−0.025 (0.024)	0.009 (0.021)
Autonomy	0.018 (0.021)	0.020 (0.021)
Support from boss	−0.063 ** (0.021)	0.178 *** (0.025)
Gender	0.029 (0.021)	0.043 * (0.022)
Age	0.007 (0.022)	−0.064 ** (0.021)
**Interaction Effect**		
Mental threat × perceived job quality and security	-	0.100 *** (0.026)
**Model Fit**		
Log-likelihood	−26,526.836	
N	2078	

Note: *β* = standardized coefficient; SE = standard error. * *p* < 0.05, ** *p* < 0.01, *** *p* < 0.001. Bootstrapped standard errors (200 replications).

## Data Availability

The dataset is secondary and publicly available, making it accessible to the general public (https://www.kosha.or.kr/oshri/researchField/downTrendsSurvey.do).
